# Maternal mortality during the COVID-19 pandemic in Mexico: a preliminary analysis during the first year

**DOI:** 10.1186/s12889-021-11325-3

**Published:** 2021-07-02

**Authors:** Nina Mendez-Dominguez, Karen Santos-Zaldívar, Salvador Gomez-Carro, Sudip Datta-Banik, Genny Carrillo

**Affiliations:** 1 Hospital Regional de Alta Especialidad de la Península de Yucatán. Subdirección de Enseñanza e Investigación, Calle 7 #433 x 20 y 22 Fracc. Altabrisa, C.P. 97130 Mérida, Yucatán Mexico; 2 Centro de Investigación y de Estudios Avanzados del IPN (Cinvestav), Department of Human Ecology, Antigua Carretera a Progreso Km. 6, 97310 Mérida, Yucatán Mexico; 3 O ‘Horan General Hospital. Hospital Epidemiologic Surveillance Unit. State of Yucatan Health Services, Avenida Itzaes s/n, Avenue Centro Jacinto Canek, 97000 Mérida, Yucatán Mexico; 4 Department of Environmental and Occupational Health, School of Public Health, Texas A&M University, 212 Adriance Lab Road, College Station, TX 77843 USA

**Keywords:** Maternal mortality, COVID-19, Pandemics, Mexico

## Abstract

**Background:**

In Mexico, the COVID-19 pandemic led to preventative measures such as confinement and social interaction limitations that paradoxically may have aggravated healthcare access disparities for pregnant women and accentuated health system weaknesses addressing high-risk patients’ pregnancies. Our objective is to estimate the maternal mortality ratio in 1 year and analyze the clinical course of pregnant women hospitalized due to acute respiratory distress syndrome and COVID-19.

**Methods:**

A retrospective surveillance study of the national maternal mortality was performed from February 2020–February 2021 in Mexico related to COVID-19 cases in pregnant women, including their outcomes. Comparisons were made between patients who died and those who survived to identify prognostic factors and underlying health conditions distribution.

**Results:**

Maternal Mortality Ratio increased by 56.8% in the studied period, confirmed COVID-19 was the cause of 22.93% of cases. Additionally, unconfirmed cases represented 4.5% of all maternal deaths. Among hospitalized pregnant women with Acute Respiratory Distress Syndrome consistent with COVID-19, smoking and cardiovascular diseases were more common among patients who faced a fatal outcome. They were also more common in the age group of < 19 or > 38. In addition, pneumonia was associated with asthma and immune impairment, while diabetes and increased BMI increased the odds for death (Odds Ratio 2.30 and 1.70, respectively).

**Conclusions:**

Maternal Mortality Ratio in Mexico increased over 60% in 1 year during the pandemic; COVID-19 was linked to 25.4% of maternal deaths in the studied period. Lethality among pregnant women with a diagnosis of COVID-19 was 2.8%, and while asthma and immune impairment increased propensity for developing pneumonia, obesity and diabetes increased the odds for in-hospital death. Measures are needed to improve access to coordinated well-organized healthcare to reduce maternal deaths related to COVID-19 and pandemic collateral effects.

## Background

Modifications in the immune and respiratory systems in pregnancy may affect the acute respiratory distress syndrome (ARDS) from COVID-19. In addition, access and availability of family planning, reproductive health, and prenatal care services have decreased due to the response to the pandemic, particularly in low and middle-income countries [1].

Regardless of the etiology, maternal mortality is an indicator of healthcare access and availability in a particular region. Mortality in pregnant women is a preventable situation and particularly deplorable since mother and child health and wellbeing are a priority in every health system worldwide [2]. When the COVID-19 pandemic reached Mexico, health resources were largely re-oriented to address it as community transmission began. As part of the strategy, specific clinics were restricted to providing only indispensable healthcare. Public hospitals were modified (reconverted) to properly reception and treat COVID-19 patients, suspending all other conventional hospital services [3].

Like other at-risk populations, pregnant women were advised to remain at home to avoid unnecessary exposure. Despite preventive measures, an unknown number of pregnant women have probably been infected with the SARS-CoV-2 virus, and a proportion of them has developed severe clinical manifestations. A new World Health Organization (WHO) epidemiologic alert was released to encourage member countries to implement measures to ensure optimal medical attention for pregnant women and promote maternal mortality prevention awareness [4].

Mexican response for COVID-19 includes telephone-based surveillance for the epidemiologic study of cases, including sampling and testing a fraction of those cases. These measures were also recommended for pregnant women, while changes to the standards of care for reproductive health, including reducing prenatal care visits, reducing postpartum consultations, caring for minor emergencies, and reducing the number of care-focused staff complemented with remote surveillance via telephone or video call. The indication for confirmed pregnant women without alarm symptoms is to stay isolated at home for 14 days or visit a second-level hospital partially or fully reconverted to treat COVID-19 if in labor or if severe respiratory syndrome occurs [5]. Like other low-middle income countries, Mexico has not yet achieved the reductions in maternal mortality recommended by the WHO since hemorrhage and hypertensive disorders during pregnancy are still associated with maternal deaths during pregnancy, labor, or puerperium [6]. Before the pandemic, for the 2019 year, the Maternal Mortality Ratio in Mexico (including direct and indirect, excluding late deaths) was 31.2 per 100 thousand births, showing a 12.8% reduction compared to the previous year; the leading causes included obstetric hemorrhage (22.5%), hypertensive disorders (20%) and underlying respiratory diseases (15%) [7]. The need for pregnant women to have periodic clinical exams, normally in healthcare environments, is an additional aggravating factor during the COVID-19 pandemic. It means they may be more exposed to contagious individuals. As the pandemic began, the public health system increasingly used telemedicine appointments to avoid potential exposure to SARS-CoV-2. However, telemedicine poses distinct challenges when used for assessing pregnant women [8].

In Mexico, a woman’s vulnerability to maternal mortality is affected by socioeconomic factors such as education, rural residence, and indigenous ethnicity [9]. All are linked to inequities in access and availability of health resources and infrastructure, which is highly variable between different states in Mexico. Based on data as of June 2020, COVID-19 morbidity and mortality in Mexico exhibited geographical variability that may correlate to interstate sociodemographic differences in health services availability and access [3]. However, the data available to date suggest that the pandemic poses serious challenges for optimally assessing maternal health in low and middle-income countries. Mexico is no exception, and additional efforts are needed to improve [10, 11].; therefore, the objectives of the present study are

1. To provide estimates of Maternal Mortality Ratio variations in Mexico, related and unrelated to COVID-19 and the pandemic in a broader sense, in 1 year nationwide.
2. Analyze the underlying health conditions and clinical course of pregnant women hospitalized due to acute respiratory distress syndrome and COVID-19.

## Methods

Retrospective surveillance of Maternal Mortality from February 28, 2020, to February 28, 2021, in Mexico in the 32 states was done with data from the General Office of Health Information. Board of Ethics of the Marist University of Merida (Universidad Marista de Merida - UMM) reviewed and approved the study design, exempting it from the need for ethical approval.

### Data sources

Three information sources were accessed. The first is the Maternal Death Bulletin, which includes weekly reports from the federal Office of Epidemiology webpage (updated February 28, 2021). These maternal mortality records derive directly from the death certificates prepared at the time of death by medical personnel. The recorded data include sociodemographic information, maternal age, the state in which the death occurred, medical insurance coverage, and the ICD codes corresponding to direct and indirect maternal mortality causes, excluding late deaths. In Mexico, maternal mortality cases are documented in health jurisdiction registries and evaluated by the Committees for Analysis of Maternal Mortality. Therefore, each case is analyzed retrospectively, and the diagnosis is ratified or rectified based on the case data. Until reviewed by a maternal mortality committee, all maternal death diagnoses are considered provisional. For consistency in the present analysis, all maternal death cases with late causes were excluded, as those pending a definitive diagnosis. Second is an epidemiological surveillance system dataset; this includes all registered cases as of February 28, 2021, and studied for COVID-19. For the present manuscript, we restricted women who were pregnant at the moment of medical assistance and were hospitalized due to ARDS, whose registries had complete information, complemented with the medical records of in-hospital attention until discharge. The third information source was the weekly report of pregnant and puerperal women understudy for COVID-19. We used to estimate the number of women who have been an understudy for COVID-19 to date proximation of lethality among those who were not hospitalized or were not pregnant but puerperal.

### Data selection

Relevant data were collected from these information sources for analysis. First, all records of pregnant women were selected, and specific data extracted from them, primarily in four categories:

(i) All patient sociodemographic data were collected. Such data included age, ethnicity, primary language (a particular note was made if they were a monolingual native speaker of an indigenous language), and their residence state.
(ii) Clinical manifestations for each patient were collected, and if they were treated ambulatorily or hospitalized, the time elapsed from symptom onset to hospitalization and presence or absence of specific signs. In addition, information of their need for intubation, admittance at the Intensive Care Unit (ICU), and general clinical evolution included the type of diagnosis established by the Mexican Health System. Such diagnosis included (PCR, Antigen test, clinically diagnosis supported by a board, Epidemiologic association with a confirmed case) including the number of days from symptom onset.
(iii) Information from in-hospital medical care, considering general characteristics of patients such as age and pre-pregnancy body mass index (BMI) indicating obesity (≥30), smoking, and the presence or absence of underlying health conditions.
(iv) Outcomes included pneumonia, intensive care unit management, and death.

### Statistical analysis

#### Maternal mortality

Nationwide and state MMRs were calculated by dividing the number of maternal deaths in the studied period by 100,000 births. The MMRs specifically attributed to COVID-19 were calculated using maternal deaths linked directly to COVID-19. Excess MMR estimates were generated by extrapolating observed MMRs to twelve months and adjusting by adding the average 10-year between-year variation. State-by-state MMRs were used to illustrate variation between MMR before and after the studied period. Calculations were also done on the percentage of maternal deaths derived from COVID-19 infections based on the total number of maternal deaths nationwide and state. Late deaths were excluded.

### Pregnant women hospitalized understudy for COVID-19

Descriptive statistics included age distribution, general information, type of sampling and diagnosis, frequency of underlying health conditions, and ARDS evolution according to their recovery/death status and within the studied timeframe. In addition, logistic regression expressing Crude Odds Ratios with binary dependent variables was used to identify associations between ARDS and underlying health conditions and outcomes (including pneumonia, intensive care unit management, and death). Statistical significance was set at *p* < 0.05; all statistical analyses were performed using Stata 15.

## Results

### Maternal mortality

**Fig. 1:**
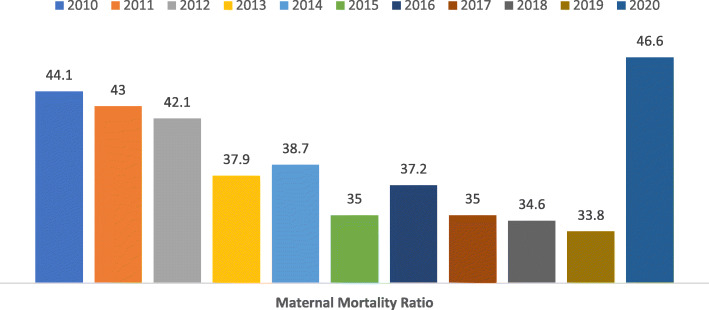
Maternal Deaths per 100,000 births in Mexico during a 10 year period, nationwide

In the studied period, 1056 maternal deaths took place, 835 reported between weeks 10 and 53, and 221 reported in the first 10 weeks of 2021. Nationwide, Maternal Mortality Ratio was 57.7 maternal deaths per 100,000 births. Between 2010 to 2019, a continuous decrease in maternal mortality was observed, with an average yearly reduction of MMR = 1.15 (Fig. 1). Therefore, for 2020 the expected MMR = 29.8, but the observed MMR = 47.2.

#### Maternal mortality ratio by state

**Fig. 2:**
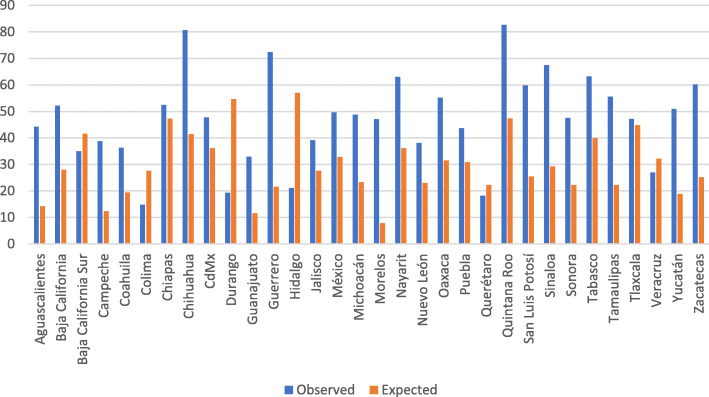
Expected versus Observed Maternal Mortality Ratio distribution among 32 Mexican States during 2020, based on 2019 registries and ten-year tendencies

This national increase of MMR between the same periods of 2019 and 2020 exhibited interstate variability (Fig. 2) with an average state increase of MMR = 16.3 (95% Confidence Intervals = 8.9–23.7).

The Maternal Mortality Ratio from COVID-19 was 13.6 (309 cases), and additionally, the unconfirmed COVID-19 Maternal Mortality Ratio was 2.4 (57 cases), representing 22.9 and 4.5% of all cases of maternal mortality in the studied period, respectively. Unconfirmed COVID-19 Maternal deaths included all ARDS cases studied as COVID-19 but remained as suspected [4].

### Hospitalizations of pregnant women with acute respiratory distress syndrome consistent with COVID-19

Between February 28, 2020, and 2021, a total of 42,525 cases of pregnant and puerperal women have been understudy due to COVID-19 infection; 33.2% (*n* = 14,137) have positive laboratory results for COVID-19, of which 299 have died, registering a lethality of 2.1%. From those cases, 7064 pregnant patients developed acute respiratory distress syndrome and received in-hospital medical assistance. The mean age of hospitalized pregnant patients was 27.7 ± 0.1 years; 133 (1.9%) were active smokers, and 637 (9.0%) had pre-pregnancy BMI ≥30, 396 Kg/m^2^ (5.6%) had underlying health conditions. (Table 1).

**Table 1:** Individual information, comorbidities and laboratory test among pregnant women hospitalized due to Acute Respiratory Distress Syndrome consistent with COVID-19, compared by recovery/mortality outcome (*N* = 7064)

	Recovery		Death	
	Mean SD		Mean SD	
Age	27.25 0.08		30.13 0.49	
	***n*** **= 6867**	**Percentage**	***n*** **= 197**	**Percentage**
< 19 or > 38 years	1308	19.05	43	21.83
19 to 38 years	5559	80.95	154	78.17
General Information				
Smoking	132	1.87	2	1.04
BMI > 30	605	8.82	32	16.41
Underlying health conditions (*N* = 396)				
Asthma	166	2.42	5	2.58
Diabetes	374	5.44	22	11.34
Immune impairment	82	1.20	4	2.06
Hypertension	363	5.29	15	7.69
Cardiovascular	46	0.67	1	0.52
Unspecified, other	367	5.39	26	13.47
Covid-19 sampling (*N* = 6853)				
PCR	6116	89.06	187	94.92
Antigen	910	13.25	19	9.64
Processed sample with positive result^a^	2289	33.33	135	68.53
Evolution of severe respiratory syndrome (7064)				
Pneumonia	1449	21.59	137	69.54
Mechanical ventilation	159	2.31	95	48.22
	Mean	Standard Error	Mean Standard Error	
Days from onset of symptoms	2.65	0.04	4.39	0.26

^a^Not all samples were processed either because (a) their samples were taken but not tested, (b) their samples were not adequate and were not repeated or (c) were determined as suspected cases and confirmed under clinical and epidemiological criteria (d) had a negative result and were not re tested. *SE* Standard Error

Time elapsed since the onset of symptoms and sampling was 2.68 ± 0.4 days. Samples for COVID-19 were from 6985 (97%) patients, of which 89 (1.3%) were diagnosed with epidemiological association with a confirmed case; 2430 (34.4%) through revision and verdict from a medical committee. Two thousand one hundred sixty-eight patients (30.7%) had a positive result (1878 (86.62%) PCR, 290(13.37%) antigen); 1414 samples (20.0%) were not processed, and 767 (10.8%) remained as suspected, either due to unreported sample (*N* = 331) or because the patient tested negative. No re-test was performed (*N* = 436). In relation to patient’s condition at discharge, of the 7064 pregnant women requiring hospitalization, 6867 (97.2%) recovered, while 197 (2.8%) faced in-hospital death. Patients who did not recover differed in age, general characteristics, underlying health conditions frequency differed between patients who recovered and patients who did not are presented in Table 1.

Regression analysis (Table 2) revealed that pneumonia was significantly associated with asthma and immune impairment. Simultaneously, patients with cardiovascular conditions and obesity were significantly more prone to be admitted to the intensive care unit. Pneumonia, diabetes, and pre-pregnancy BMI > 30 significantly increased the odds for in-hospital death.

**Table 2:** Association between underlying health conditions in pregnant women and outcomes including Pneumonia, Intensive care unit management and death among hospitalized due to Acute Respiratory Distress Syndrome consistent with COVID-19 (*N =* 7064)

	Crude Odds Ratio	Standard Error	Z	*p*	Confidence Interval	
**Pneumonia** ***N =*** **1586**						
Smoking	1.11	0.23	0.51	0.609	0.74	1.67
Diabetes	1.08	0.14	0.56	0.575	0.83	1.40
**Asthma**	**1.66**	**0.29**	**2.85**	**0.004**	**1.17**	**2.34**
BMI > 30	1.20	0.12	1.84	0.066	0.99	1.46
**Immune impairment**	**1.62**	**0.39**	**2.00**	**0.045**	**1.01**	**2.60**
Hypertension	1.23	0.16	1.63	0.103	0.96	1.59
Cardiovascular	1.06	0.37	0.16	0.872	0.53	2.09
Chronic kidney Disease	1.20	0.43	0.51	0.611	0.60	2.41
**Intensive Care Unit** ***N*** **= 478**						
Smoking	2.50	0.94	2.43	0.810	1.19	5.25
Diabetes	0.91	0.27	−0.30	0.581	0.51	1.64
Asthma	1.32	0.55	0.67	0.592	0.59	2.98
**BMI > 30**	**1.17**	**0.26**	**0.69**	**0.010**	**0.75**	**1.81**
Immune impairment	1.26	0.67	0.43	0.114	0.44	3.60
Hypertension	1.45	0.41	1.32	0.310	0.83	2.53
**Cardiovascular Disease**	**1.77**	**1.16**	**0.87**	**0.019**	**0.49**	**6.42**
Chronic kidney Disease	1.39	1.00	0.46	0.281	0.34	5.65
**Death** ***N =*** **197**						
Smoking	0.48	0.35	−1.01	0.313	0.12	1.98
**Diabetes**	**2.30**	**0.57**	**3.35**	**0.001**	**1.41**	**3.73**
Asthma	0.99	0.46	−0.01	0.991	0.40	2.47
**BMI > 30**	**1.77**	**0.38**	**2.71**	**0.007**	**1.17**	**2.69**
Immune impairment	1.50	0.79	0.77	0.444	0.53	4.23
Hypertension	0.94	0.29	−0.21	0.832	0.51	1.72
Cardiovascular	0.63	0.65	−0.45	0.655	0.08	4.72

## Discussion

We have presented the MMR estimates increase during the pandemic and provided an overview of the magnitude of COVID-19 confirmed and unconfirmed maternal mortality cases. In addition, when analyzing pregnant women hospitalized due to COVID-19, we have identified particular conditions that increased vulnerability for adverse outcomes.

Of the 1056 maternal deaths recorded in Mexico in 1 year, COVID-19 was to some extent associated with 27.5% of maternal deaths that occurred. During this period, it was the leading cause of maternal death in the country. Before the advent of COVID-19, maternal mortality’s leading causes were obstetric hemorrhages and hypertensive diseases [11, 12]. This nationwide MMR was surpassed in twelve states; the most dramatic increases were observed in Guerrero, Morelos, and Chihuahua.

In the present study, the Maternal Mortality Ratio due to COVID-19 was 13.6 for confirmed and 2.4 for unconfirmed cases, while lethality among hospitalized pregnant patients was 2.8%. These results are far above the projected panorama of more than COVID-19-related maternal deaths for the United States of America [13]. A systematic review of thirteen studies from three countries (China, the United States of America, and Italy) found mortality among pregnant women with COVID-19 to be zero [14]. In other studies, maternal mortality is not assessed, for instance, in a metanalysis review of adverse pregnancy outcomes among pregnant women with COVID-19 in China and European countries [15]. The present results suggest that in Mexico, COVID-19 among pregnant women may have an unprecedented fatality rate.

Access to proper and timely care during pregnancy can dramatically affect outcomes. Intensive care unit (ICU) access is particularly vital in acute COVID-19 cases, but this can vary widely between different countries. In a study mentioned above, [14] ICU admissions of pregnant women with COVID-19 in China, the United States of America, and Italy averaged 3%. In a study of pregnant women with COVID-19 (*N* = 637; 1.6% mortality) hospitalized in China, ICU admission was 9.6% [16], higher than the present study’s 1.7% ICU admission rate. The second cause of maternal mortality in Mexico during the study was hypertensive diseases, proteinuria in pregnancy, puerperium (15.1%), and obstetric hemorrhage (13.8%). These disorders may be unrelated to COVID-19 infection per se. Still, the conditions prevailing in Mexico during the pandemic (e.g., long-term confinement and its immediate social correlates) may be propitious for allowing treatable adverse conditions to end in maternal mortality. This situation warrants further analysis since adequate prenatal control helps identify women with risk factors for developing obstetric hemorrhages, such as clinical diagnosis of the accrete placenta. However, the massive increase in demand for health services caused by the COVID-19 pandemic in Mexico has severely taxed the public health system’s capacity. The need for more ICU wards and additional healthcare personnel to treat the influx of COVID-19 cases resulted in the implementation of strategies such as suspension of chronic and non-urgent care in hospitals [17–20] and giving ICU priority to COVID-19 cases.

Maternal mortality is an indicator of a country’s development level. It evidences poverty and social exclusion, and in 2015, WHO reported that 303,000 women died due to complications during pregnancy or birth, which translates to an MMR of 216 deaths per 100,000 live births. Of these deaths, 99% occurred in developing countries, and most were preventable [20].

In the current environment, this is unattainable in Mexico, which is particularly alarming compared to the 39 deaths per 100,000 live births from all causes of maternal mortality nationwide from 2005 to 2014 [21].

Maternal mortality has increased in Mexico due to COVID-19, specifically in severe cases manifesting ARDS and/or pneumonia. Complications were more probable when the cardiorespiratory system was affected. The particular vulnerability of pregnant women to SARS-CoV-2 can be explained by the probable involvement of the pathophysiological pathway involving T-helper system response and th1/th2 balance, as described for the Influenza A virus [22]. Individual lifestyle may also have some effect on case severity. The way to address this threat is for maternal health care professionals to closely monitor patients with COVID-19. Their clinical manifestations need to be controlled using a preventive approach focusing on those with underlying chronic conditions, which documents signs and symptoms of severity as a way of deciding if and when ambulatory or in-hospital treatment is indicated. Procedures need to be developed based on available resources and existing guidelines, and patients need to be aware of them [23, 24].

As shown in the present results, not all maternal deaths in Mexico are directly related to COVID-19 infection but rather to uncontrolled conditions during pregnancy due to the limited healthcare availability. Social distancing and limited mobility are crucial to reducing SARS-CoV-2 transmission. Nonetheless, healthcare during pregnancy is also essential to ensure healthy pregnancies. In-person medical attention can be reinforced during this pandemic by telemedicine or web-based strategies to monitor and detect any pregnancy or COVID-19 related complication and guide decision-making, including suitable transportation to specialized health centers [25].

Unintended pregnancies are commonly associated with late or no prenatal care in Mexico [26], and these pregnancies may be more frequent at both extremes of the reproductive age span (< 18 or > 38 years). During the pandemic, unintended pregnancies can be complicated by confinement and consequent delays in or omission of early medical attention. A recent estimate is that 145,719 births in Mexico in 2020 were from unintended pregnancies, mainly in response to the social behaviors derived from confinement and the barriers to adequate healthcare and family planning created by the pandemic [27]. For instance, the Pan-American Health Organization (PAHO) recently called on member states to intensify and improve access to prenatal care services and preventive measures to reduce COVID-19 morbidity and mortality. Furthermore, the PAHO asked its members to renew their commitment to reducing maternal and perinatal mortality even more than pre-pandemic [4], implying an urgent need for preventive strategies at all levels.

### Limitations

The retrospective design applied in the study poses limitations such as the inability to add relevant information (e.g., more specific clinical manifestations, pregnancy duration, or fetal/neonatal health status). Such information could be relevant when explaining Mexico’s maternal mortality panorama during the current pandemic. Data on pre-pregnancy BMI > 30 was collected at the moment of COVID-19 diagnosis. The patient’s social, economic, and reproductive determinants could not be retrieved for the present study because these data are not available until the annual datasets are completed and published. Furthermore, it was not within the study scope to identify increases in mortality due to COVID-19 because it did contemplate access to women with high-risk pregnancies in healthcare facilities.

## Conclusion

The MMR in Mexico increased over 60% in 1 year during the pandemic; COVID-19 was linked to 25.4% maternal deaths in the studied period. Lethality among pregnant women with a diagnosis of COVID-19 was 2.8%, and while asthma and immune impairment increased propensity for developing pneumonia, obesity and diabetes increased the odds for in-hospital death. Measures are needed to improve access to coordinated well-organized healthcare to reduce maternal deaths related to COVID-19 and pandemic collateral effects. The results highlight the need for timely, organized, and efficient maternal and obstetric healthcare at all times, but particularly during a pandemic. Providing pregnant women access to the necessary healthcare resources in the community and clinical settings must be considered a priority.

## Data Availability

The datasets generated and/or analysed during the current study are available in the Genny Carrillo and Nina Mendez ResearchGate repository, DOI: 10.13140/RG.2.2.16853.14567

## Abbreviations

ARDS: Acute Respiratory Distress Syndrome
ICU: Intensive Care Unit
MMR: Maternal Mortality Ratio
OR: Odds Ratio
PCR: Polymerase Chain Reaction
